# Methodology and Implementation of a Randomized Controlled Trial (RCT) for Early Post-concussion Rehabilitation: The Active Rehab Study

**DOI:** 10.3389/fneur.2019.01176

**Published:** 2019-11-08

**Authors:** Johna K. Register-Mihalik, Kevin M. Guskiewicz, Stephen W. Marshall, Karen L. McCulloch, Jason P. Mihalik, Martin Mrazik, Ian Murphy, Dhiren Naidu, Shabbar I. Ranapurwala, Kathryn Schneider, Paula Gildner, Michael McCrea, Active Rehab Study Consortium Investigators

**Affiliations:** ^1^Department of Exercise and Sport Science, Matthew Gfeller Sport-Related Traumatic Brain Injury Research Center, University of North Carolina at Chapel Hill, Chapel Hill, NC, United States; ^2^Injury Prevention Research Center, University of North Carolina at Chapel Hill, Chapel Hill, NC, United States; ^3^Department of Epidemiology, University of North Carolina at Chapel Hill, Chapel Hill, NC, United States; ^4^Division of Physical Therapy, Department of Allied Health Sciences, School of Medicine, University of North Carolina at Chapel Hill, Chapel Hill, NC, United States; ^5^Faculty of Education, University of Alberta, Edmonton, AB, Canada; ^6^Canadian Football League, Toronto, ON, Canada; ^7^New Zealand Rugby, Wellington, New Zealand; ^8^Faculty of Kinesiology, Sport Injury Prevention Research Centre, University of Calgary, Calgary, AB, Canada; ^9^Cummings School of Medicine, Alberta Children's Hospital Research Institute, Hotchkiss Brain Institute, University of Calgary, AB, Canada; ^10^Acute Sport Concussion Clinic, Sport Medicine Centre, University of Calgary, Calgary, AB, Canada; ^11^Department of Neurosurgery, Center for Neurotrauma Research, Medical College of Wisconsin, Milwaukee, WI, United States

**Keywords:** traumatic brain injury, exercise, clinical intervention, post-concussion activity, return to play

## Abstract

**Background:** Sports-related concussion (SRC) is a complex injury with heterogeneous presentation and management. There are few studies that provide guidance on the most effective and feasible strategies for recovery and return to sports participation. Furthermore, there have been no randomized studies of the feasibility, safety, and efficacy of early rehabilitation strategies across multiple sports and age groups. This international cluster-randomized pragmatic trial evaluates the effectiveness of early multi-dimensional rehabilitation integrated with the current return to sport strategy vs. the current return to sport strategy alone.

**Methods:** The study is a cluster-randomized pragmatic trial enrolling male and female athletes from 28 sites. The sites span three countries, and include multiple sports, levels of play (high school, college, and professional), and levels of contact. The two study arms are Enhanced Graded Exertion (EGE) and Multidimensional Rehabilitation (MDR). The EGE arm follows the current return to sport strategy and the MDR arm integrates early, MDR strategies in the context of the current return to sport strategy. Each arm employs a post-injury protocol that applies to all athletes from that site in the event they sustain a concussion during their study enrollment. Participants are enrolled at pre-season baseline. Assessment timepoints include pre-season baseline, time of injury (concussion), 24–48 h post-injury, asymptomatic, and 1-month post-injury. Symptoms and activity levels are tracked post injury through the return to play process and beyond. Injury and recovery characteristics are obtained for all participants. Primary endpoints include time to medical clearance for full return to sport and time to become asymptomatic. Secondary endpoints include symptom, neurocognitive, mental status, balance, convergence insufficiency, psychological distress, and quality of life trajectories post-injury.

**Discussion:** Outputs from the trial are expected to inform both research and clinical practice in post-concussion rehabilitation across all levels of sport and extend beyond civilian medicine to care for military personnel.

**Ethics and Dissemination:** The study is approved by the data coordinating center Institutional Review Board and registered at clinicaltrials.gov. Dissemination will include peer-reviewed publications, presentation to patients and public groups, as well as dissemination in other healthcare and public venues of interest.

**Clinical Trial Registration:** www.ClinicalTrials.gov, identifier: NCT02988596

**Trial Funding:** National Football League.

## Introduction

Concussion is a complex injury. Athletes who sustain a sports-related concussion (SRC) present with a diverse array of symptoms and recovery trajectories (1). Unfortunately, there is limited empirical evidence for clinicians to use in selecting the most effective and feasible strategy for recovery, rehabilitation, and return to sport. Currently, return to activity recommendations are based on expert consensus, with relatively few randomized controlled studies directly evaluating return to sport strategies (2). There are few clinically directed and pragmatic options to guide clinicians responsible for implementing concussion treatment/rehabilitation, particularly during the early acute/sub-acute presentation phase. A conservative strategy of restrictive physical and cognitive rest (i.e., removing athletes from participation and placing him/her on rest until normal brain functioning returns), was long considered to be the preferred therapeutic option for athletes post-concussion and was endorsed as the standard of practice by expert panels (3). This strategy is often frustrating for athletes, given they tend to be physically-focused, task-orientated individuals. In fact, recent evidence suggests that strict, total rest may actually prolong functional recovery following concussion (4). Over the past 5 years the evidence base concerning active management and rehabilitation strategies for concussion has significantly grown and suggests various interventions may be beneficial, especially in athletes with prolonged symptoms (5–7) However, it remains unclear to what extent these active strategies can be employed without negatively affecting recovery (e.g., exacerbating symptoms, prolong symptom recovery, etc.). There is scientific and clinical concern that prematurely implementing overly-aggressive activities has the potential to worsen symptoms and delay return to activity in athletes (8, 9).

There are limited data promoting a systematic approach to early rehabilitation and post-concussion activity that is modifiable throughout the return to sport process based on symptom presentation and sport specific requirements. Young adults with cervicogenic and vestibular symptoms experiencing prolonged concussion symptoms demonstrated improved outcomes and accelerated recovery when engaged in targeted therapy to address these dysfunctions (10–12). Aerobic exercise within a symptom limited heart rate range also improves recovery and outcomes in individuals with prolonged symptoms (6, 13). However, such interventions may not consider other areas such as balance or visual disturbance and have not been fully evaluated in the context of the current return to sport paradigm in a pragmatic field setting. While some of these studies were published after the current trial protocol development, they serve as evidence for the need to further evaluate various intervention methods post-concussion, even today.

To date, no studies have addressed key and focused strategies that can be feasibly implemented at a low cost and with few resources early in the treatment process. Furthermore, no studies have prospectively evaluated the current return to sport strategy and direct integration of early, multifaceted activities into this paradigm. Additionally, no studies to date have developed a comprehensive strategy for providers to begin engaging athletes with clinically directed and symptom-based activities immediately following the recommended (14) 24–48 h rest period. Such studies are needed for application across a wide variety of sports medicine and clinical settings. In order to develop best practices for the safe and effective use of these new therapies, there is a need for **pragmatic field trials** to support the accurate development of guidance for the use of early, active rehabilitation therapies, relative to current practice.

To address this gap, we are conducting a pragmatic cluster-randomized trial with two parallel groups. Of note, the trial was designed in 2016 and the outcomes and interventions selected were based on the following factors most relevant and applicable at that time: (1) common data elements in large-scale concussion studies (15); (2) pragmatic assessments and exercises that would apply in a variety of settings and that do not require extensive resources; and (3) logical intersection with the current return to sport paradigm. The trial includes athletes of varying age and levels of skill, from multiple countries, from multiple sports, and across multiple care models to understand the influence of early activity in the context of the return to sport strategy on outcomes following sport-related concussion. The two Specific Aims for this trial are to: (1) evaluate the effectiveness of the enhanced graded exertion (EGE) progression (current return to sport strategy) vs. an early, activity rehabilitation [multidimensional rehabilitation (MDR)] strategy; and (2) evaluate the safety and feasibility of these protocols.

## Methods and Analysis

### Overview and Structure of the Active Rehab Study

The Active Rehab Study Consortium was initially proposed in 2014 through an international meeting that included representation from the scientific community and sporting organizations. The core idea of a multi-sport, multi-age, and multi-country study evaluating treatment and management of concussion was refined into a formal protocol over a period of months by an executive research consortium. They titled this project “Role of Active Rehabilitation in Concussion Management: A Randomized Controlled Trial (The Active Rehab Study).” The final consortium, led by The University of North Carolina at Chapel Hill and Medical College of Wisconsin, includes collaborators and sites from the Canadian Football League (CFL, 9 team sites), New Zealand Super Rugby (NZR, 5 team sites), North American Colleges/Universities (6 school sites), and Wisconsin and North Carolina High Schools (8 school sites). Sports represented in the study include collision, contact, and non-contact sports for both males and females. Should professional cohort sample size not approximate anticipated numbers, an additional professional ice-hockey cohort may be included. The study is conducted in compliance with US and international guidelines for research under the primary protocol approval from The University of North Carolina at Chapel Hill Institutional Review Board. All participants provide written and informed consent prior to participation. Informed consent documentation is verified via an informed consent tracking form in the study data collection system and through communication with study cohort leads and sites throughout the course of the study.

### Allocation to Study Arm

The two treatment arms (multidimensional active rehab and EGE progression) are assigned at random to the 28 sites in the study. Site level (cluster) randomization is utilized because the study team considered that patient-level randomization at a site would be prone to contamination between arms. Thus, all athletes at a given site receive the same protocol. All study sites are randomized to either the MDR (early rehabilitation) or the EGE [current return to sport strategy (16)]. To ensure a balanced of treatment arms across cohort, site randomization is stratified by (i.e., conducted within) cohort (NZR, CFL, College, HS). Colleges/universities and high school sites are stratified by size of school prior to randomization; CFL and NZR sites are not stratified. Due to the nature of the early and active treatment delineation, no allocation concealment such as masking or blinding is possible. The clinicians (site personnel) at the sites know their allocated arm. However, participants are not explicitly told about the role of their respective study arm. Site personnel at the MDR sites are trained to deliver the treatment separately from the site personnel at the EGE sites.

### Participants and Eligibility Criteria

The inclusion criteria for participants in the trial are individuals rostered as an athlete at the study sites who consent to the study. Written, informed consent is administered during a pre-season baseline assessment. Target participant enrollment across all settings is estimated to be 3,500 at baseline and 100–200 in each study arm (total *n* = 200–400) post-injury. The post-injury protocol includes all consented athletes with a SRC at each site and meeting the following criteria.

Our current trial aligns with common elements from the NCAA-DOD Grand Alliance Concussion Assessment, Research, and Education (CARE) Consortium (15). As such, we have defined SRC in accordance with the Department of Defense (DoD) operational definition as *a change in brain function following a force to the head, which may (or may not) be accompanied by temporary loss of consciousness (if LOC, temporary is defined as* <*30 min based on the Mayo TBI severity guidelines), but is identified in awake individuals with measures of neurologic and cognitive dysfunction, as indicated by 1 or more of the 22 symptoms from the Sport Concussion Assessment Tool (SCAT) symptom checklist* (16). No athlete with a Glasgow Coma Scale <13 enters the treatment progression of either arm.

As is standard with SRC studies (15), identifying the SRC is determined by medical professionals at each site involving a physician and other team-based healthcare provider (based on clinical exam and their interpretation of objective findings inclusive of the definition above). If medically diagnosed with a SRC, and no other indicators of more moderate to severe TBI as defined in the Mayo definition above (17), consented participants are eligible for enrollment in the treatment protocol. Documentation of the clinical diagnosis and identification of the medical personnel making the diagnosis are recorded in study case report forms. For inclusion in the post-injury protocols, the SRC must occur in a rostered sport for a high school or collegiate sport at their school or for their specific rostered sport (and team sanctioned activity) for the professional cohort. Individuals with any positive/abnormal clinical neuroimaging finding(s) following injury are not entered into the post-injury protocol or are discontinued from their arm treatment protocol if these findings are observed after the protocol has been initiated. Although these individuals are discontinued from the treatment protocol, we continue to collect assessment time point data on these individuals and documentation for their overall care.

We anticipate ~10–15% attrition due to study demands and seasonal nature of sport through full clearance to return to sport. However, we expect 20–30% attrition for the 1-month timepoint due to this timing and other potential participant follow-up issues. The study protocol incorporates contacting participants to keep them engaged.

### Study Arms and Treatment Protocols

**Figure 1:**
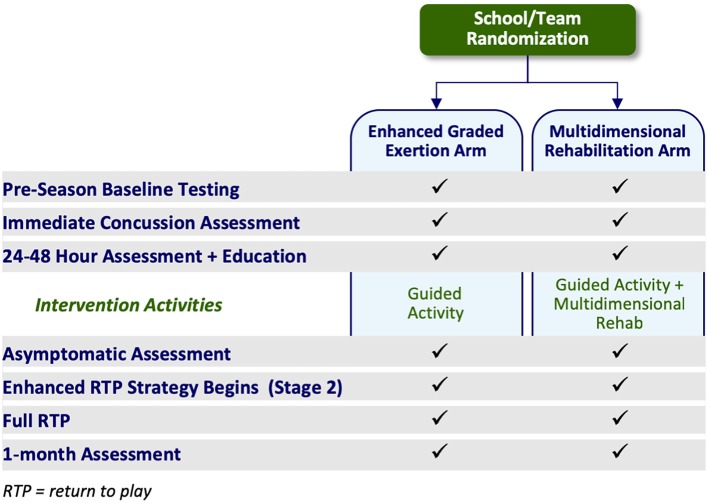
Key study activities by study arm.

The two study arms are EGE and MDR. The EGE arm primarily follows the current consensus return to sport progression (Table 1). The MDR arm includes early, active rehabilitation that is integrated into the EGE/return to sport progression. Overall, the difference between study arms is the inclusion of early, active rehabilitation (Figure 1).

**Table 1:** 5th International Consensus Statement on concussion in sport return to sport strategy.

**Rehabilitation Stage**	**Functional exercise at each stage of rehabilitation**	**Objective of each stage**
**1. Symptom-limited activity**	Daily activities that do not provoke symptoms	Gradual reintroduction of work/school activities
**2. Light aerobic exercise**	Walking or stationary cycling at slow to medium pace. No resistance training	Increase HR
**3. Sport-specific exercise**	Skating drills in ice hockey, running drills in soccer. No head impact activities.	Add movement
**4. Non-contact training drills**	Progression to more complex training drills (e.g., passing drills in football and ice hockey). May start progressive resistance training	Exercise, coordination, and increased thinking
**5. Full contact practice**	Following medical clearance, participate in normal training activities	Restore confidence and assess functional skills by coaching staff
**6. Return to play**	Normal game play	

*From McCrory et al. (16) 5th International Consensus Statement on Concussion in Sport (NOTE: Our study was designed prior to the 2016 strategy being released, however, in anticipation of Stage 1 changing to limited/symptom guided activity, our design always included that as part of Stage 1 and the remainder of the strategy remained the same)*.

Participants in both arms, and at all sites, are enrolled into the overall study at pre-season baseline. However, the site's treatment protocol is only activated following concussion injury. Specifically, the post-injury protocol for both arms is only activated if the consented athlete suffers a concussion related to their rostered sport of interest at a team sanctioned event and meet enrollment criteria post-injury.

Following activation of the protocol post-injury, all concussed participants are given guidance on recommended physical activities in which they can engage. This guidance is consistent with the 5th International Consensus Statement on Concussion in Sport (16). Of note, our study was designed prior to the 2016 strategy being released, however, in anticipation of Stage 1 changing to limited/symptom guided activity, our design always included this type of language as part of Stage 1, as well as more generic descriptions of each stage. Participants are also instructed by their site medical staff on how to be observant for increases in symptoms. This guidance—which focuses on guided activity rather than restriction—is provided via a hardcopy educational instruction sheet and a short video. These materials are provided to all participants following injury. All concussed participants also keep a daily physical and cognitive activity summary log from 24 to 48 h post-injury through 7 days post return to play. A small subset of participants wears activity tracking technology to track physical activity from time of injury to full return to play. There is no predetermined sample size for the activity trackers as this is an ancillary component only. The activity log information serves as the primary compliance measure, as well as measures of activity that may affect recovery (covariates). To enhance compliance for log completion, participants are sent email reminders where applicable and completion is monitored by site clinicians for all sites.

#### Guided Rest + Enhanced Graded Exertion (EGE Arm)

Participants in the EGE arm complete the activities described above and guided rest prior to progressing past Stage 1 of the graded exertion (Table 1) (16). The term EGE was chosen as sites are directed to be sports specific in their choice of activities throughout the progression. A medical professional determines the symptom status of the athlete and when Stage 2 of the graded exertion for return to sport will begin. Once the athlete has been asymptomatic for 24 h (within at least 85% of their baseline symptom score—definition of asymptomatic for the study) they may begin the EGE progression. This protocol follows the 5th International Consensus Statement on Concussion in Sport return to sport strategy (16), but encourages enhancement to include sports and skill specific activities. Each step is recommended for completion on a separate day, at the clinician's discretion. Clinicians complete session logs for each graded exertion session for Stage 2 and for subsequent stages that include the following information: initial symptom checklist, phase of graded return to play progression, specifics on session activities, percentage of rest during the session, participants rating of perceived exertion, final symptom checklist, session satisfaction rating, and overall session feedback.

#### Guided Rest + Multidimensional Rehabilitation + Enhanced Graded Exertion (MDR Arm)

**Figure 2:**
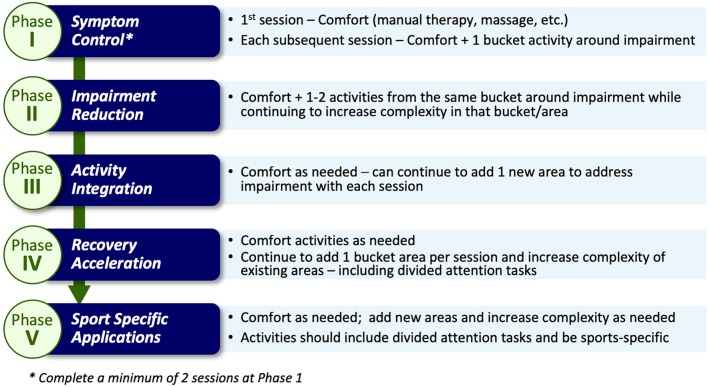
Multidimensional Rehabilitation (MDR) framework and progression.

The term MDR was chosen to illustrate more than one area of activity would/could be addressed. Participants in the MDR arm complete the same activities as the EGE arm participants (as described above). However, once the participants' symptoms become “stable” (i.e., not getting worse), they are progressed into the MDR activity phases. “Stable” is defined as no significant increase utilizing Reliable Change Indices (RCI) metrics [symptom score not increasing by 10 or more over a 24-h period from their initial (first) symptom assessment] and no significant development of new symptoms over 24 h. Prior to beginning the exercises in the intervention, clearance to do so is obtained and documented by the athlete's healthcare team. The intervention includes 5 progressive phases: symptom control, perceived impairment reduction, activity integration, recovery acceleration, and sport specific application (Figure 2).

**Figure 3:**
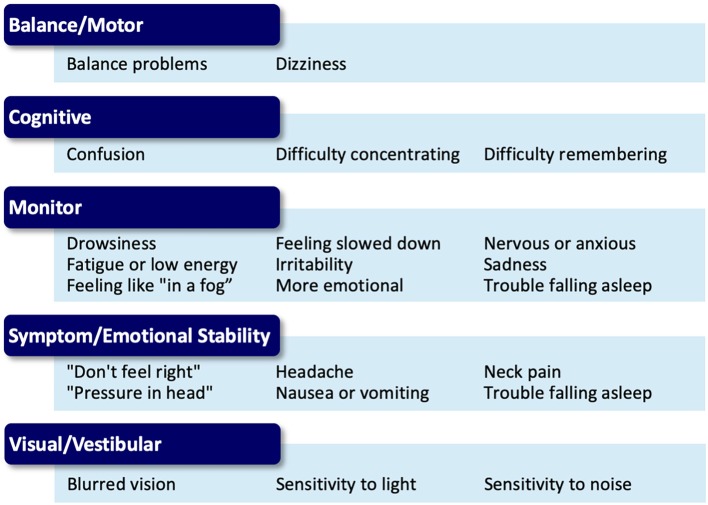
Symptom presentation and activity bucket matches for the Multidimensional Rehabilitation (MDR) progression.

The choice of activity type in each phase is dependent on the nature of the athlete's reported symptoms and noted assessment deficits via a symptom assessment and clinical interview at each phase. Once an athlete is asymptomatic these activities may be chosen based on sport-specific performance needs. Activities are grouped into categories (termed “buckets”) that are matched to a participant's symptom reports (Figure 3). The activity “buckets” include: balance, cognitive, comfort (symptom/emotional stability), and visual-vestibular. Some symptoms do not necessarily match the activity buckets and should be monitored. Activities that meet the intensity of targeted buckets are selected by the clinician, and are extensively documented, similar to the documentation process utilized by Schneider et al. (12).

Active, MDR sessions consist of guided exercises directed by a team clinician. Participants are asked to complete four sessions per week until full return to play, at their healthcare team's discretion. During each session, clinicians complete session documentation logs that include the following information: initial symptom checklist, phase of graded return to play progression, specifics on session activities, percentage of session spent resting, participants rating of perceived exertion, and final symptom checklist, session satisfaction rating, and session feedback. Should a participant state they are feeling worse during a session or request to stop, symptoms will be immediately assessed by the clinician. Sessions are stopped if a participant exceeds reliable change on total symptom severity (10 or more total point increase) (18, 19), if the participant requests to stop, or if the provider feels the participant is too symptomatic to continue. The symptom scale utilized is the SCAT 22-item (each item scored 0–6) post-concussion symptom scale. The metric utilized from this is total symptom burden (severity), which is calculated by summing the score of each item for a possible score range of 0–132.

Progression through the MDR protocol follows a standardized set of rules (Table 2). Progression from Phase I (Symptom Control) to Phase II (Perceived Impairment Reduction) requires that an individual's symptoms must not increase 10 or more points compared to their lowest symptom assessment since injury, and they must not have any symptoms with a symptom score of 5 or 6 at the beginning of a subsequent intervention session. When the participant completes activities in the phase where symptoms remain stable/do not increase beyond reliable change from beginning of one session to beginning of another (see “stable” above), the participant will be progressed to the next phase. We expect some increase from beginning to the end of a session, but we expect this to decrease by the start of the next session. Participants should on average, complete four sessions per week until fully returned to play. One session each week may be completed at home (i.e., unsupervised) as directed by a team healthcare provider. Each session lasts ~20 min and is conducted at the clinician's discretion. Once enrolled into the MDR study arm, each participant completes a minimum of two sessions in Phase I (Symptom Control). The MDR activities may commence prior to beginning the EGE progression and should be integrated with EGE activities once a participant is asymptomatic.

**Table 2:** Multidimensional rehabilitation progression (Active Rehab).

**Rehabilitation stage**	**Notes**	**Goal**
**Entry into Phase I** (to the intervention progression)	*Symptoms not getting worse*. ∘ Symptom score not increasing by 10 or more over a 24 h/1 day period from their initial symptom assessment (6 h or 24–48 h assessment) ▪ Most people will be eligible at this time ▪ The earliest someone could start the intervention would be 24–48 h post-injury	Stabilization of symptoms
**Phase I**	*Must complete a minimum of **2 sessions** in this phase* Progression to Phase I may occur when: ∘ An individual's symptoms must **not** increase 10 or more points **compared to their lowest symptom score since the injury** ∘ They must not have any individual symptom items with a severity score of **5 or 6** when assessed at the beginning of a subsequent intervention session at Phase II (this would be the third session or beyond)	Symptom control and introduction to the intervention
**Phases II–III, Phases III–IV, and Phases IV–V**	*Must be a minimum of 1 day spent at each phase. Two phases cannot be completed on the same day*. Progression from one of these phases to the next (2–3, 3–4, and 4–5) may occur when: ∘ An individual's total symptom severity score does **not** increase by 10 or more points from beginning of one intervention session to beginning of the subsequent session ∘ No individual symptom item severity score symptom score at the beginning of the subsequent intervention session is a **5 or 6**	Phase II- Perceived Impairment reduction Phase III- Activity integration Phase IV- Recovery acceleration Phase V- Sport specific application
**Considerations for care and progression** Clinicians should consult their site medical team regarding issues of pre-existing conditions and presentation that may affect care. Some of these may include:		
Migraine headaches Sleep related conditions/symptoms Gross vestibular dysfunction		
Documentation of all additional care and treatment should be completed including but not limited to:		
Medications Additional therapies (e.g., physical therapy, vision therapy, vestibular therapy, etc.) All referral sources and those involved in the individual's care		
**Return to full participation**		
Return to full participation will occur at the physician/site medical professional's discretion of patient full recovery and will be documented. The intervention progression will stop.		

**Figure 4:**
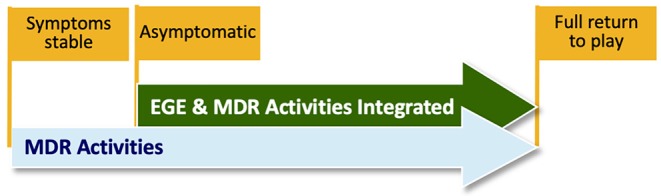
Example illustration of Multidimensional Rehabilitation (MDR) activities being integrated to the Enhanced Graded Exertion (EGE) progression. This figure illustrates the overlap in activities. MDR activity may be integrated as soon as the participant is asymptomatic.

**Figure 5:**
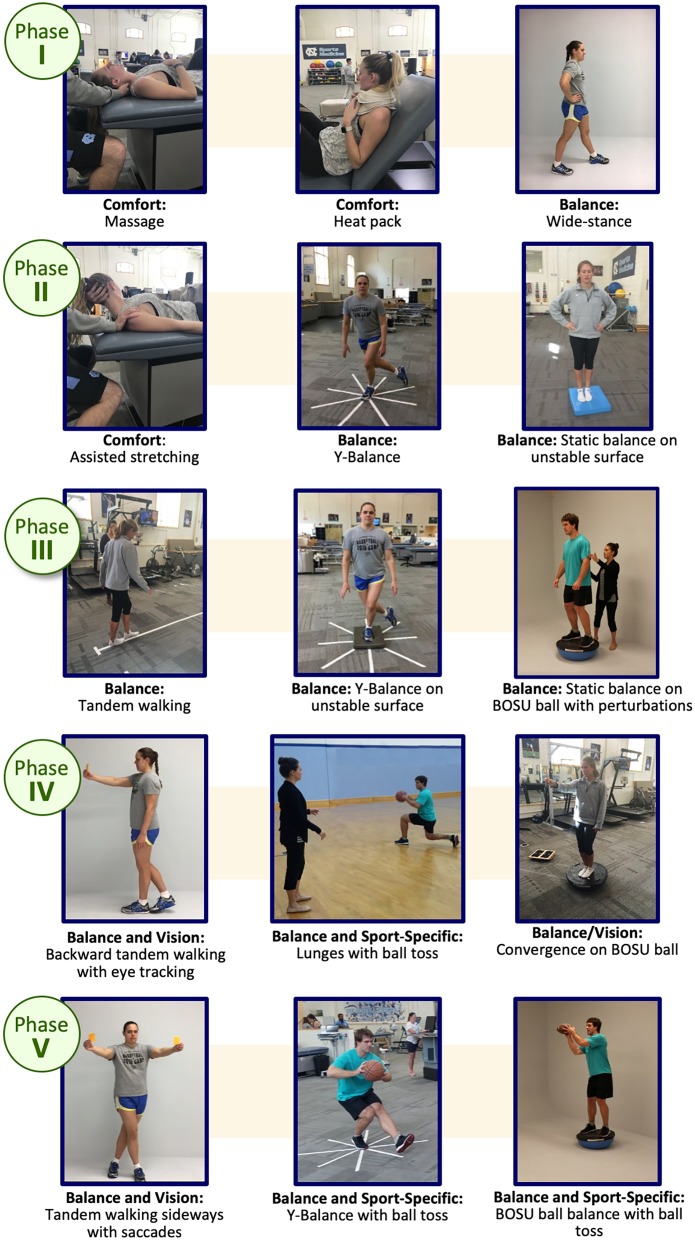
Example Balance Activity Progression through the Multidimensional Rehabilitation (MDR) framework. Written informed consent was provided by all individuals in the images for publication.

Specifically, progression from one phase to the next in Phases II through V requires that the participant does not experience any significant increase in symptoms from the beginning of one intervention session to the beginning of the subsequent session (as measured by a RCI of 10 of more total severity point increase), and no symptom score at the beginning of an intervention session is a 5 or 6 on the self-reported symptom severity scale. Once determined to be clinically recovered (“asymptomatic” by study definition or at clinician discretion), they begin the EGE progression (16) (Table 1) with sport and skill specific enhancements at each phase. This MDR protocol should not delay the return to play process as when the participant becomes “asymptomatic” (by the study definition) they begin the enhanced graded return protocol (as the standard of care states) and will continue MDR exercises throughout this process. MDR activities are integrated with the return to sport progression at each Stage once the return to sport progression begins (Figure 4). Participants continue the rehabilitation progression during the EGE protocol and may continue the MDR exercises after full return for maintenance and/or to complete the last phases of the MDR progression at their clinician's discretion. Figure 5 provides an example of cognitive activity progressions through each Phase. The Supplemental Table 1 outline activity “bucket” progressions by MDR phase.

### Safety Procedures

As with any trial, safety-related procedures were decided *a priori* by the study team in concordance with current literature. Potential risk of the study assessments and interventions were evaluated. Based on previous studies and clinical fortitude, it was determined *a priori* that discomfort is likely (10–25%) to occur as the participants are progressed in the interventions. We specifically anticipated more discomfort among those in the multidimensional activity group, as discomfort may increase slightly during rehabilitation sessions, as often occurs within any type of rehabilitation session. All rehabilitation is monitored and progressed by medical professionals and individuals are referred to their team physician as deemed medically appropriate. If at any point the participant or clinician feels the intervention should be discontinued, this is done and documented. Injury risk is rare, however, there is the small possibility that symptom exacerbation or injury may occur during the interventions or testing. As all participants will be studied and progressed in environments with medical professionals, any potential significant symptom exacerbation or injury is documented, and participants are referred to the physician at the institution as deemed appropriate. The physician and medical team at each site will determine status and ability to continue the study activities. All events of this nature are documented appropriately via study administrative forms. A symptom-based adverse event was determined to be, as outlined in the progression, if an individual's symptoms increased by a reliable change of 10 or more points and remained elevated at that change in the subsequent session. An independent safety officer reviews quarterly study safety reports provided by the study team study and provides feedback on any overall concerns or safety issues. If the safety officer deems the study unsafe after corrective actions have been put into place, the study will be halted, or significant changes may be made to the study methods.

### Data Management

All data are managed on secure servers through the data coordinating site via a central database or through site-based collection measures. All participants are registered with an identification code. Source data includes any original documentation to the study. The database is monitored by the data coordinating site and kept current to ensure monitoring of data and appropriate follow-up of participants throughout the study protocol. Monthly, quarterly, and individual injury monitoring occurs across the entire study period by the data coordinating site to ensure data quality and timely entry.

### Study Outcomes and Assessments

The primary trial endpoints include time to asymptomatic/symptom free and time to full clearance for return to sport, in days. The secondary endpoints include clinical and quality of life outcomes assessed from baseline through 1-month post return to play, as well as safety and feasibility outcomes. An assessment protocol similar to the NCAA-DOD Grand Alliance Concussion Assessment, Research, and Education (CARE) Consortium's is utilized for both baseline measurements and post-injury assessments and to achieve the primary and secondary endpoints (15). The assessment time-points for the study (Table 3) include: pre-season baseline, time of injury (optional, 24–48 h following injury, daily symptom and activity tracking through 7 days post-return to play, athlete satisfaction at 7 days post-return to play, and 1-month following full return to play. Each of the assessment timepoints are collected at the approximate windows, i.e., within 5 days, due to the nature of athletic schedules. Study measures are administered by trained site personnel and clinicians. Assessments take place at site medical and training facilities. Below are brief descriptions of all study measures.

**Table 3:** Assessment schedule.

	**Demographics**	**Personal and family history**	**SCAT symptom checklist**	**QOL**	**BSI-18**	**Neurocognitive assessment**	**SAC**	**BESS**	**NPC**	**Dual-task**	**Start and end fatigue rating**
**Pre-season baseline**	✓	✓	✓	✓	✓	✓	✓	✓	✓	✓	✓
**Time of injury (within 6 h—if possible)**			✓				✓	✓			
**24–48 h post-injury**			✓		✓	✓	✓	✓	✓	✓	✓
**Asymptomatic post-injury**			✓	✓	✓	✓	✓	✓	✓	✓	✓
**1-month post return to play post-injury**			✓	✓	✓	✓	✓	✓	✓	✓	✓
- Symptoms and activity assessed daily from the first assessment point until 7 days post return to play - Concussion Index completed following injury - Recovery Form completed following return to play - *Participant Satisfaction completed at 7-days post return to play*											

*QOL, Quality of Life; BSI-18, Brief Symptom Inventory 18 item; SAC, Standardized Assessment of Concussion; NPC, Near Point of Convergence; SCAT, Sport Concussion Assessment Tool*.

**Demographics**: Demographic information is collected on a separate form depending on the study cohort (i.e., High School, College/University, and Professional Setting). This assessment includes standard demographic information such as date of birth, sex at birth, place of birth, and race. In addition, information regarding sports history and academic level/achievement will be collected. *Time point collected: Baseline*.

**Concussion History**: The concussion history form provides the participant with a definition of concussion prior to asking the participant to provide a self-report of concussion history. Participants are directed to a concussion summary report for each concussion they report to have experienced. In the summary report the participant is asked to identify whether the concussion was sport-related or not, if the concussion was diagnosed, the approximate date of injury, their age at the time of injury, whether or not they lost consciousness (for how long), if they experienced any form of amnesia, and the number of days they experienced symptoms related to this particular concussive injury. *Time point collected: Baseline*.

**Medication History**: The medication history form requires the participant to identify any prescription medications she/he is currently taking as well as any over the counter medications. Prescription medications are broken up into categories (antidepressants, anti-psychotics, narcotics, non-narcotic pain medication, sleep aids, psychostimulants, birth control, allergy medication, asthma medication, and medication for acid reflux). The participant is asked to identify the exact name of any type of medication they are currently using. Three over the counter medications are listed for the participant to identify using including ibuprofen, acetaminophen, and loratadine. The participant is given space to identify any other over the counter medications they are currently using that are not listed. Lastly, the participant is asked to identify any supplements they may be using, and to report their tobacco, marijuana, and alcohol use. *Time point collected: Baseline*.

**Medical History**: The medical history form contains questions regarding the following self-reported information: height, weight, handedness and headache history. Participants are also asked about diagnosis of the following: meningitis, seizures, diabetes, sleep disorders, balance disorders, vestibular disorders, vertigo, motion sickness, Meniere's disease, psychiatric disorders, and other conditions. Participants are asked to provide information regarding previous diagnosis of conditions such as: learning disorders, attention deficits, hyperactivity disorder, vision and hearing issues, stroke, Parkinson's, and memory disorders. Participants also report any family history of headaches, migraines, Parkinson's, and memory disorders. Lastly, participants are asked to report their sleep patterns. *Time point collected: Baseline*.

**Symptomology**: The Standardized Concussion Assessment Tool symptom checklist (3, 16) includes a 22-item symptom inventory, self-reported hours of sleep inquiry, and questions regarding factors that may influence the severity of a participant's symptoms (i.e., mental/physical activity). Each participant is asked to rate how they feel “on a normal day” at baseline and “now” post-injury, with respect to each particular symptom, on a 6-point scale ranging from “none to severe” (0–6, respectively). Reliability and validity of the symptom checklist is well-established (20). Each symptom item score is added together to determine overall symptom burden (symptom severity score); higher scores indicate greater symptom burden (severity). For this study, a reliable change is considered as a change of 10 points or more (18, 19). The possible score range for burden is 0–132. (*Time points collected: Baseline, Time of Injury, 24–48 h post injury, Asymptomatic, and 1-month post return to play. Note that participants who are injured and enter into the post-injury protocol are also asked to complete symptom checklists at the beginning and end of each intervention session*.

**Brief Symptom Inventory-18 (BSI-18):** Psychological distress is measured utilizing the BSI-18. The BSI-18 is a brief symptom inventory with high reliability (21). The assessment gathers athlete-reported data to help measure psychological distress in primary care settings (21). Participants rate their level of distress associated with 18 symptom items on a scale from 0 (not at all) to 4 (extreme). Ratings are then added together to compute an overall symptom distress score. *Time points collected: Baseline, 24–48 h Post-Injury, Asymptomatic Post-Injury, and 1-month post return to play*.

**Health-Related Quality of Life (HRQL**): Participants' HRQL is assessed using the Athlete-Reported Outcomes Measurement Information System (PROMIS-29), and the Quality of Life in Neurological Disorders (Neuro-QOL) Cognition and Fatigue Scales. These scales have high reliability and validity concerning overall quality of life (22–24). Outcomes will include the PROMIS-29 and Neuro-QOL summary scores (anxiety, physical function, depression, sleep disturbance, social role/activities, pain interference, pain intensity, Neuro-QOL cognition, and Neuro-QOL fatigue). These scales ask the participant to rate items on a Likert type scale ranging from 1 to 5 (higher or lower score indicating “worse” is dependent upon the item). *Time points collected: Baseline, Asymptomatic, 1-month post return to play*.

**Computerized Neurocognitive Testing**: Participant neurocognitive performance at baseline and post-injury will be assessed utilizing the computerized neurocognitive testing platform currently used clinically at each site. The platforms to be included by study sites include Immediate Postconcussion Assessment and Cognitive Test (ImPACT), CogSport, and Concussion Vital Signs. Reliability and validity of computerized tests varies and has been established in previous literature (25, 26). Each platform includes alternating forms and presentation variation. *Time points collected*: *Baseline, 24–48 h post injury, Asymptomatic, and 1-month post return to play*.

**Mental Status**: The Standardized Assessment of Concussion (SAC) (varied forms) (27, 28) will be used to assess mental status. The SAC is a clinical measurement to determine an individual's cognitive orientation, concentration ability, and immediate/delayed memory recall. The SAC has been shown to be a reliable and sensitive measure of concussion (27, 28). Alternate forms are used at each time point. *Time points collected: Baseline, Time of Injury, 24–48 h post injury, Asymptomatic, and 1-month post return to play*.

**Figure 6:**
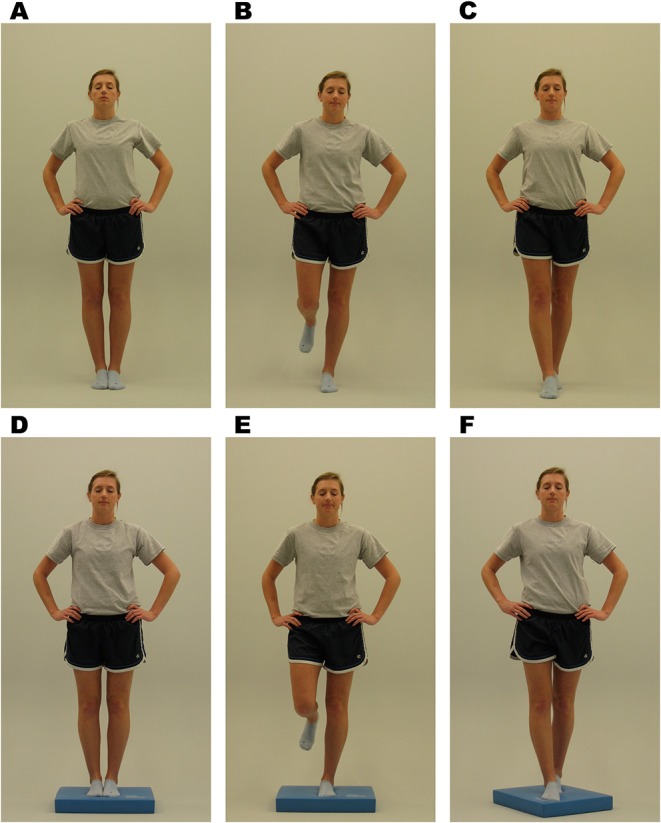
Balance Error Scoring System (BESS) stances. Written informed consent was provided by the individual in the image for publication. **(A)** Double leg firm, **(B)** single leg firm, **(C)** tandem stance firm, **(C)** double leg foam, **(E)** single leg foam, and **(F)** tandem stance foam (29).

**Balance**: Balance is assessed utilizing the Balance Error Scoring System (BESS), as it is an objective postural stability measure that can be implemented in an office, field, or clinic setting. The test is administered as the participant completes three 20 s stance trials (i.e., double leg, single leg, tandem stance) on firm and foam surfaces (Figure 6). The administrator tracks errors during the trials. The BESS has been shown to have high reliability and sensitivity and specificity (30). The outcome from the BESS will be the total error score. *Time points collected: Baseline, Time of Injury, 24–48 h post injury, Asymptomatic, and 1-month post return to play*.

**Near Point Convergence**: The Near Point of Convergence (NPC) test will function as the visual/oculomotor exam for this study (31). NPC is measured by drawing a tongue depressor with a dot in 14-point font from arm's length toward the participant's nose. The participant is instructed to stop the approximation at the point the visual target is seen in double (diplopia). The clinician then measures the distance between the tip of the nose and the tongue depressor in centimeters. Three trials are collected and averaged. NPC has been shown to be a reliable measure (32). *Time points collected: Baseline, 24–48 h post injury, Asymptomatic, and 1-month post return to play*.

**Figure 7:**
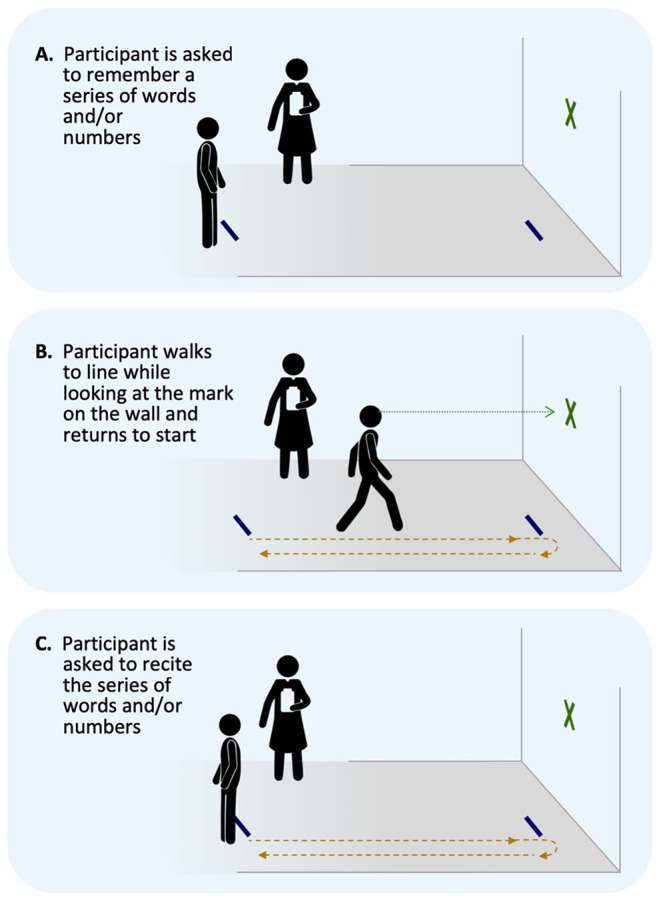
Dual-task schematic. **(A)** Memory task begins, **(B)** walking task, and **(C)** memory recall.

**Dual-task**: Dual-task performance will be assessed via the Walking and Remembering Dual-Task Assessment (ISAW-Grid Task) (33) which has been previously reported as potentially useful for physically active individuals (34) and evaluates divided attention cost across a gait and cognitive task. Participants walk 3.5 m toward a target and then turn and walk back to the start line and the walk time is recorded. Participants are then given 2 numbers and 6 letters from the military phonetic alphabet and asked to recall the information accurately and in order. Alternate word list are given at each time point. Following these single tasks, the individual is then asked to combine the task. The examiner gives the individuals 2 numbers and 6 letters to remember, the participant completes the gait task, and upon return to the start line, the participant is asked to recall the numbers and letters (Figure 7). Gait time and accuracy are scored and coded as the initial outcomes. Performance in the dual-task is then compared for each of these outcomes to the single task, yielding the primary outcome of dual-task cost. Participants will be asked to complete a cognitive (immediate recall) task while also completing a walking task of 7 m. *Time points collected: 24–48 h post injury, Asymptomatic, and 1-month post return to play*.

**Daily Activity and Symptom Tracking**: All concussed participants complete a daily activity and symptom tracking survey (cognitive and physical) from time of injury through 7 days post return to play. The survey can be completed on paper or via Qualtrics and includes questions about mental and physical activity as well as symptoms. *Time point collected: Daily through 7-days post return to play*.

**Participant Satisfaction**: The participant will be asked to complete a questionnaire regarding his/her satisfaction with the rehabilitation sessions and the intervention. This measure is adapted from the PSQ-18 which is a publicly available scale that measures general athlete satisfaction with care. The PSQ-18 was designed based on feedback from athletes using input from providers about the care they receive and has been used in various settings. *Time point collected: 7 days post return to play*.

**Concussion Injury Index**: This form documents all aspects of a participant's concussion. It is completed by the clinical research staff at the study site. Information gathered on this form includes: sport at time of injury, number of years playing sport, date and time of injury, date reported injury, loss of consciousness, etc. *Time point collected: This form is to be completed over the course of the injury and should be finalized by the 1-month post-return to play time point*.

**Recovery Tracking**: A recovery tracking form documents all aspects of a participant's recovery from a concussion. It is completed by the clinical research staff at the site throughout the time it takes the participant to recover. Length of symptoms, medication usage, therapies/treatments, psychiatric issues, and return to play information will be reported. It also includes information on completion or discontinuation of the study treatment activities (e.g., discontinuation of the intervention for medical reasons). *Time point collected*: *This form should be completed over the course of the injury and should be finalized by the time entry is completed for the 1-month post-injury assessment*.

### Planned Data Analysis

We conduct quarterly, interim analysis for descriptive outcomes to determine continued safety and feasibility of the study and to prepare safety reports.

***Specific Aim 1*** *(evaluation of effectiveness):* For analyses of our primary endpoints, Cox proportional hazards regression models (35) will be used to compare time to return to play and time to asymptomatic between the EGE and MDR groups. The specific outcomes for the Cox models will be time from date of injury to: (1) date of medical clearance for full return to participation and (2) asymptomatic date. The Wei-Lin robust variance estimator will be used to account for the effect of cluster-randomization by site (36).

For our secondary endpoints (clinical and quality of life measures), recovery trajectories will be examined by use of General Linear Mixed regression models and non-parametric smoothers. Random effects will be utilized to account for the effect of clustering by site and the effect of repeated observations over time within an individual participant. The time axis to be modeled in both sets of analyses is time from initiation of the treatment (defined as stable symptoms for 24 h), and time will be treated as a continuous variable in all analyses.

We will also assess potential predictors of attrition (e.g., gender, race, socioeconomic status). If no predictable patterns are observed for missing data (i.e., missingness occurs at random), no imputations will be conducted. Inverse probability of attrition weights based on the factors influencing attrition will be used to account for potential selection bias due to attrition. We will assess the differences between the EGE and MDR group participants (those with SCR) at baseline and before starting the treatment protocol.

For sensitivity analyses of primary and secondary, we will conduct intent-to-treat analysis (prescribed treatment), per-protocol analysis (adhered treatment) and use inverse probability weighting to determine potential outcomes had everyone adhered to their prescribed treatments.

To test the effectiveness of randomization, we will compare key variables at baseline and immediately post-injury (24-48 h timepoint) to determine differences between arms. These variables at a minimum will include: age, gender, previous history of concussion, contact/collision sport, baseline symptom severity score, 24–48 h symptom severity score. Should any differences be observed these factors will be controlled for in the models. Additionally, 24–48 h symptom severity will be considered in all analyses.

***Specific Aim 2*** (safety and feasibility): We will utilize descriptive statistics, qualitative analyses for open ended text of perceptions (exploratory based; triangulation) to understand overall safety, adverse event prevalence, and protocol perceptions.

***Sample size*** (determined based on primary outcomes): Given that each participant will be recruited between 6 and 48 h post-concussion and will be followed for a month after their return to play (average total time of ~37 days), if we estimate that each arm will have at least 100 participants, we will have 83% power to estimate an effect size of 0.64 in the MDR group as compared to the EGE group concerning days to asymptomatic. Table 4 shows the available power for varying sample and effect sizes.

**Table 4:** Power and effect size based on number of participants in each arm.

	**Hazard ratio for MDR vs. EGE comparing time to asymptomatic**				
*Randomized arm size*	*4/7 (0.57)*	*4.5/7 (0.64)*	*5/7 (0.71)*	*5.5/7 (0.79)*	*6/7 (0.80)*
100	95%	83%	60%	36%	18%
150	>99%	94%	78%	50%	24%
200	>99%	98%	88%	62%	30%
250	>99%	>99%	94%	72%	37%

## Discussion

A major success of the study thus far is the international collaboration between researchers and clinicians across multiple collision sports and competitive levels in exchanging ideas regarding the understanding early rehabilitation for SRC and the current return to sport paradigm. This multidisciplinary collaboration engineered strategic solutions for the challenges encountered in implementing a large pragmatic randomized controlled trial. This seamless collaboration is critical to the successful launch and execution of the Active Rehab Study.

### Varied Models of Clinical Care

Basic models of SRC care differ with varied settings across several countries, sports, and competitive levels. In the US, Athletic Trainers are commonly engaged and are often the primary clinicians delivering the intervention. In Canada, Athletic Therapists are most commonly the frontline providers directing care. In New Zealand, physicians and physiotherapists are the providers who deliver the intervention. Within these medical structures, there are differences in the standard protocol based on the site's overarching sport governing body (e.g., National Collegiate Athletic Association, Canadian Football League, World Rugby, High School Federation, etc.). The Active Rehab Study protocol, while prescriptive, also allows for clinical decision-making to ensure practical application and implementation on a larger scale. Funding to support front-line staff across these care models is also important and considering how this funding may be implemented locally is also a key factor for success. Additionally, given that many participants are professional athletes, it is important for leagues, schools, and administrators to understand that participants' medical providers are still responsible for their medical care and return-to-play decision-making to ensure compliance with the study trial. Without allowing site-specific medical oversight, many of the sites agreeing to participate in our study would have declined.

### Changing Landscape of SRC Management

Implementing a multiyear pragmatic clinical trial involves understanding the rapidly changing landscape of SRC management. With a rapidly growing evidence base and new treatment and management strategies emerging, it is important to provide a protocol to capture any of these adaptations that may occur in clinical care across the trial. Our trial does not prohibit additional care and clinical decisions outside of the study protocol due to these potential changes. As such, we capture all treatments and activities outside of the study protocol to be able to control and assess how these factors may influence our study outcomes. Additionally, as the study began, the 5th International Consensus Statement on Concussion in Sport (16) had not yet been released. However, we felt symptom limited activity during Stage 1 was often clinically practiced vs. no activity. As such, this has been our protocol from the beginning of the study.

### Clinical Variability of SRC

SRC presents in various ways and often involves an individualized approach. As such, it is important that the protocol allow for clinical-decision making within the context of the protocol. Additionally, participants may present with other symptoms or signs of medical conditions needing additional treatment. As such, allowance for additional treatments are a necessary part of a study like the current trial. Activities outside of the study protocol are closely documented to be able to control and assess how these factors may influence study outcomes.

### Data Collection, Integrity, and Analysis

Quality assurance is a top priority to ensure maximum rigor of methods and confidence in the results of the study. Integrity of data collection and study arm/intervention documentation is an ongoing process that includes initial trainings for sites and onboarding of clinicians who will administer assessments and/or interventions. Yearly refreshers for those continuing with the study in multiple years are provided either in-person or via video training. Additionally, clear, concise, and specific study manuals for each aspect of the study are available to all study sites and team members, but are arm specific for the intervention portions. Post-injury checklists, specific to the study arm are available to all sites to ensure each participant follows the designated protocol and subsequent study specific activities in his/her arm. The coordinating institution is notified of an injury to ensure the study protocol steps are followed. Additionally, while there is a central study data system, one cohort utilized an application that collected the data and these data are merged with the larger array of data. The data systems all meet security requirements for the various institutions with individual password access and tracking. All data entry mechanisms contain data type and value range limitations to control for extraneous data entry. Monthly, quarterly, and injury specific monitoring occur by the project manager and project coordinator to ensure timely and accurate collection and entry. Following these monitoring mechanisms, sites are notified of issues with corrective actions and asked to correct and notify the data coordinating site when corrections have been made. These corrections are then verified by the data coordinating center. Data are cross-checked for quality within the monitoring system and via the quarterly preliminary analysis exports. Quarterly detailed data checks are run for standard distributions, missingness, and detailed data quality. Sites may be asked to further review and verify data with the oversight of the project manager to correct data through this mechanism. Additionally, in-person meetings and trainings are conducted to build relationships, answer questions about the study, and promote data quality and study success.

### Intervention Compliance

Due to the interventional nature of both study arms, a high level of intervention compliance and documentation of activities during the rehabilitation and return to play process is essential. As described above, regular training, study manuals, and monitoring are key to ensuring site compliance and corrective actions when deviations occur such as missing study assessment timepoints and incorrect post-injury rehabilitation or return to play progressions. Additionally, having a clinician coordinator who manages the SRCs at various levels of sport being the primary point of contact for the rehabilitation (MDR) and return to sport (EGE) progressions continues to be essential to increase clinician buy-in and compliance. To increase athlete compliance, the sessions are clinician guided and include activities important to the participant.

### Limitations and Future Considerations

As with any trial, the current protocol does not include every potential treatment area for concussion. However, the intent of the trial is to address key areas of concerning in a patient-centered and pragmatic manner in an effort to translate findings to a variety of clinical settings. Future trials and evaluation work may consider additional domains or assessment strategies as well as utilizing these assessments in areas of progression in more targeted populations and settings in which more clinical time and capacity may be available.

### Anticipated Outcomes

Outcomes from the ongoing trial will contribute to research efforts to better understand the effects of early rehabilitation and the current return to sport paradigm on recovery time. Research efforts like this ongoing trial provide a pragmatic framework for research that seeks to produce the highest-level evidence possible concerning management and treatment of SRC across sports and across various levels of play in differing medical care environments. Lastly, the study data will provide guidance to safely and effectively use early, active rehabilitation therapies in the current clinical landscape. In order to achieve these outcomes we expect to see a positive effect of the MDR and that both arms will illustrate both MDR and EGE to be safe and feasible. We anticipate then being able to develop implementation manuals and strategies to be used in a variety of clinical settings.

## Ethics and Dissemination

The study is carried out in accordance with international standards of research and under the guidance of the data coordinating center Institutional Review Board. Additional approvals and reviews are conducted as necessary for all study sites. Consideration is given to local needs and cultural considerations in the ethics review and implementation process. Written, informed consent is provided by each participant. For minors in the high school cohorts, guardian consent is also obtained. The trial is registered at clinicaltrials.gov (NCT02988596). Participants are enrolled at pre-season baseline to provide an opportunity to consent prior to a concussion occurring and reduce respondent burden post-injury. The model consent form may be obtained from the corresponding author upon request. All intervention and study activities occur with site medical professional guidance. Site investigators and clinicians explain the study protocol to participants are available to participants for questions and concerns. After completion of the trial and statistical analysis of the trial data, findings will be published in peer-reviewed medical journals and will adhere to CONSORT standards. The current plans for these primary papers include: (1) a primary paper addressing effectiveness of the intervention arms on the primary outcomes of time to clearance for full return to sport and time to asymptomatic, (2) a paper addressing effectiveness of the interventions on the secondary clinical outcomes, (3) a paper concerning overall safety and symptom provocation for both intervention arms, and (4) a paper addressing implementation evaluation and feasibility of the study interventions. Additional dissemination of results will include presentation at relevant scientific meetings, to the general public, and through peer-reviewed publications.

## Ethics Statement

The study was approved by the Institution Review Board at the University of North Carolina at Chapel Hill (and associated site ethics boards where needed) and is registered as a clinical trial (clinicaltrials.gov; NCT02988596). Prospective participants are fully informed of the procedures of the study. Informed consent (and guardian consent) is provided by all participants prior to engaging in any study activities. Reporting procedures are in place to ensure any serious adverse events are report to the Principal Investigator.

## Author Contributions

JR-M, KG, MMc, and SM contributed to study design, data analysis and interpretation, initial drafting, and revision as well as final approval of the manuscript. KM, JM, MMr, IM, DN, KS, and PG contributed to study design, revision, and final approval of the manuscript. SR contributed to study design, data analysis, revision, and final approval of the manuscript. The Active Rehab Consortium investigators contributed to data collection, revision, and final approval of the manuscript.

### Conflict of Interest

The authors declare that the research was conducted in the absence of any commercial or financial relationships that could be construed as a potential conflict of interest. The handling editor declared a past co-authorship with one of the authors KS.

## Active Rehab Study Consortium Site Investigators

Canadian Football League and the University of Alberta: Marty Mrazik, Ph.D., R.Psych, Dhiren Naidu, MD, FRCPC, Dip. Sport Med. New Zealand Rugby: Ian Murphy, BHB, MBChB, MMedSci, FACSEP, MBA, Danielle Salmon, Ph.D. Catawba College: Brandon Auton, BS, ATC. Elon University: Caroline J. Ketcham, Ph.D., Eric E. Hall, Ph.D. University of Lynchburg: Thomas G. Bowman, Ph.D., ATC, Caroline A. Wesley, MS, ATC. North Carolina Central University: Elizabeth Jewell, MA, ATC. York University: Loriann Hynes, Ph.D., CAT(C), R.Kin.
