# Supplementary material for: Methodology and Implementation of a Randomized Controlled Trial (RCT) for Early Post-concussion Rehabilitation: The Active Rehab Study
Source: Front Neurol. 2019 Nov 8;10:1176. doi: 10.3389/fneur.2019.01176 (PMC6856221; doi:10.3389/fneur.2019.01176)
Supplement: Supplementary file 1 [file Table_1.DOCX]

**SUPPLEMENT. Example Activities for the Multidimensional Rehabiltiation Progression by Phase**

| **Comfort & Cervicogenic** | **Phase 1** | **Phase 2** | **Phase 3** | **Phase 4** | | | **Phase 5** |
| --- | --- | --- | --- | --- | --- | --- | --- |
|  | **Symptom Control** | **Impairment Reduction** | **Activity Integration** | **Recovery Acceleration** | | | **Sport Specific** |
| **Sample Dose Per Phase 🡪** | **5-10 Minutes** | **5-10 Minutes** |  |  | | |  |
| **Activity** | | | | | | | |
| **Range of Motion/ Gentle Stretching: Cervical Spine** | All ranges:  Passive and or active | All ranges:  Passive and or active |  | |  |  | |
| ** PLEASE NOTE **  Athletes must be able to tolerate AROM prior to initiating any activities involving head movement (nodding, turning, etc.) Ex. Vestibular ocular reflex (VOR) activities | | |  | |  |  | |
| **Soft Tissue Massage** | Cervical/upper thoracic musculature | Cervical/upper thoracic musculature |  | |  |  | |
| **Trigger Point Release** | Cervical/ upper thoracic musculature | Cervical/ upper thoracic musculature |  | |  |  | |

| **Cognitive** | **Phase 1** | **Phase 2** | **Phase 3** | **Phase 4** | | | **Phase 5** |
| --- | --- | --- | --- | --- | --- | --- | --- |
|  | **Symptom Control** | **Impairment Reduction** | **Activity Integration** | **Recovery Acceleration** | | | **Sport Specific** |
| **Sample Dose Per Phase 🡪** | **1-2 sets, 1-2 min** | **2-3 sets, 1-2 min** | **2-3 sets, 1-2 min** | **3 sets, 2-3 min** | | | **3 sets, 2-3 min** |
| **Activity** | | | | | | | |
| **Simple Math or Digit Span** | Count to 100 by 7  *In a quiet environment | Basic addition/ subtraction problems *In a quiet environment | Either of the previous activities  *In a busy environment | | Either of the previous activities with added vision task (ex. Convergence) | Either of the previous activities with added sport specific vision task (ex. Ball toss visual tracking) | |
| **COWAT** | Identify a category (ex. animals, cars, fruits, etc.) Athlete names as many as they can within specified timeframe | Identify a more difficult category (ex. States and Capitals, school and mascot, all the orange fruits and veggies you can think of, etc.) | A simple list but in a different language if they know one or are familiar with one (ex. colors, numbers, food, etc.) | | COWAT plus eye tracking activity (ex. near/far, left/right, light ball toss, etc.) | Sport Specific: players on the team, plays, etc. (ex. Name all the players on the team in order of seniority…) with a balance task | |
| **Memory and Attention** | Name two objects for every letter in your first/ last name | Look around your environment, find 5 red objects that can fit in your pockets, and 5 blue objects that are too big to fit | Increase the number of objects named in either of the previous examples.  *No repeats with the first sample activity if name includes 2 of the same letter | | Either of the previous activities with an added static/ dynamic balance task | Either of the previous activities with an added sport specific balance task | |
| **Imagery** |  |  | Game situation, imagery or questions *Running a specific play in FB, what’s your route? Or BB runner on 2^nd^, two outs, ball hit to you, where do you go… | | Game situation, imagery or questions * In a wall sit | Game situation, imagery or questions * Added balance Task: Ready position on BOSU | |

| **Visual/Vestibular** | **Phase 1** | **Phase 2** | **Phase 3** | **Phase 4** | | | **Phase 5** |
| --- | --- | --- | --- | --- | --- | --- | --- |
|  | **Symptom Control** | **Impairment Reduction** | **Activity Integration** | **Recovery Acceleration** | | | **Sport Specific** |
| **Sample Dose Per Phase 🡪** | **2-3 sets,**  **30 sec- 1 min** | **2-3 sets,**  **30 sec- 1.5 min** | **3 sets,**  **1- 1.5 min** | **3 sets,**  **1- 1.5 min** | | | **3 sets,**  **1- 1.5 min** |
| **Activity** | | | | | | | |
| **Smooth Pursuits/Eye Tracking:**  **Horizontal/Vertical Directions** | Eye Tracking: Patient seated, object arms-length away, neutral background *One direction | Eye Tracking:  Standing *Double leg balance, firm or foam | Eye Tracking:  added busy background/ environment | | Eye Tracking: added busy background or wall sits | Sport specific tracking:  Ex. volleyball: Low level/ stationary pepper focused on tracking the ball | |
| **Saccades:**  **Horizontal/Vertical Directions** | Patient seated, looks back and forth between 2 points about arms-length away  *One direction | Have patient complete standing or add a direction | Patient completes previous task with a different background (ex. colored wall, stripes/ patterns, etc.) | | Hand-eye/foot-eye coordination task: light ball toss during a balance activity focusing on tracking the ball | Sport specific tracking:  Ex. volleyball: Low level/ stationary pepper focused on tracking the ball | |
| **Convergence** | Pencil pushups: Seated | Pencil pushups: Standing | Pencil near/far:  Patient looks back and forth between pencil (close to nose) and object in distance | | Patient completes previous task with added wall-sits or balance activity | Sport Specific tracking:  Near/ far focus activity  Ex. Basketball: Free throw shots | |
| **VOR:**  **Horizontal/Vertical Directions** ** PLEASE NOTE** that an athlete must be able to tolerate AROM prior to initiating any VOR activities | Patient seated, holds object arms-length away, patient moves head in specified direction while maintaining gaze on object | Have patient complete standing or add a direction | Add one of the previously mentioned progressions, or add a busy background (ex. different colored wall, stripes, patterns, etc.) | | Have patient complete exercises in a wall-sit or in a balance position | Sport-specific hand-eye or foot-eye coordination drills (require similar head movement): Ball toss | |

| **Balance** | **Phase 1** | **Phase 2** | **Phase 3** | **Phase 4** | | | **Phase 5** |
| --- | --- | --- | --- | --- | --- | --- | --- |
|  | **Symptom Control** | **Impairment Reduction** | **Activity Integration** | **Recovery Acceleration** | | | **Sport Specific** |
| **Sample Dose Per Phase 🡪** | **2-3 sets 30 sec - 1 min, eyes open** | **2-3 sets 30 sec -1.5 min, eyes open or closed** | **3 sets 1-1.5 min, eyes open or closed** | **2-3 sets 1-1.5 min, eyes open or closed** | | | **3 sets 1.5 – 2 min eyes open or closed** |
| **Activity** | | | | | | | |
| **Gait** | Double leg stance, single leg stance, tandem stance | Double leg stance, single leg stance, tandem stance, Tandem gait *Unstable surface, foam, disc, etc. | Tandem gait *Backwards/sideways | | Tandem gait *Backwards/ sideways, with added visual task: smooth pursuits, saccades, VOR | Tandem gait *Backwards/ sideways, with added visual task: smooth pursuits, saccades, VOR | |
| **Y-Balance** | Y-balance reaches, Ant., Post lat, post med  *Choose one or two directions | Y-balance reaches, Ant, post/lat, post/med  *All directions | Y-balance reaches, Ant, post/lat, post/med  *Arms reach/ arm motion or,  **Slide board or unstable surface | | Previous activities with added cognitive task  *Attention/memory task | Sport Specific reactive task  Ex. Baseball pitcher: Specify a balance position required of the pitching motion (on stance or lead leg)  *Added head movement to mimic quick fielding or quick throw to first | |
| **Lunges** | Balance in wide stance, hands on hips | Stationary Lunges, hands on hips | Walking Lunges: Stationary or with a twist, Hands on hips | | Walking Lunges along strait line, hands on hips  *Added cognitive task, COWAT | Walking Lunges along straight or jagged path (backwards) with added sport specific reactive task *Equipment: balls, handheld sport specific equipment, etc. | |
| **BOSU** |  | BOSU: Clocks or around the world | BOSU: Clocks or around the world *With or without perturbations | | BOSU: With ball toss to encourage eye tracking and head movement | Ready position on BOSU  with perturbations or sport specific hand-eye/foot-eye coordination task *Attention or memory task | |

| **Dual Task** | **Phase 1** | **Phase 2** | **Phase 3** | **Phase 4** | | | **Phase 5** |
| --- | --- | --- | --- | --- | --- | --- | --- |
|  | **Symptom Control** | **Impairment Reduction** | **Activity Integration** | **Recovery Acceleration** | | | **Sport Specific** |
| **Sample Dose Per Phase 🡪** |  |  |  | **2-3 sets 1-2 min** | | | **2-3 sets 1-3 min** |
| **Activity** | | | | | | | |
| **Cognitive** |  |  |  | | Digit Span + Lunges with a twist | Digit Span + VOR exercise | |
|  |  |  |  | | COWAT + balance activity | COWAT +low intensity/ stationary ball handling drills | |
|  |  |  |  | | Simon + Tandem Gait | Game situations + Sport Specific Balance Task | |
| **Visual/Vestibular** |  |  |  | | Convergence on the BOSU ball | Convergence on the BOSU ball (Around the world) | |
|  |  |  |  | | VOR + 100 by 7 | VOR + 100 by 7 (In a wall sit) | |
|  |  |  |  | | Ball Toss + Rocker Board or BOSU (In ready position) | Vestibular activity + Balance Activity | |
| **Balance** |  |  |  | | Balance foam pad + VOR | Balance Lunge + VOR | |
|  |  |  |  | | BOSU + Digit Span (With perturbations) | BOSU + hand-eye coordination activity in ready position (ball toss) | |
